# Genome-Wide Identification of Circular RNAs in Response to Low-Temperature Stress in Tomato Leaves

**DOI:** 10.3389/fgene.2020.591806

**Published:** 2020-11-05

**Authors:** Xuedong Yang, Yahui Liu, Hui Zhang, Jinyu Wang, Gaurav Zinta, Shangbo Xie, Weimin Zhu, Wen-Feng Nie

**Affiliations:** ^1^Shanghai Key Laboratory of Protected Horticulture Technology, The Protected Horticulture Institute, Shanghai Academy of Agricultural Sciences, Shanghai, China; ^2^Department of Horticulture, College of Horticulture and Plant Protection, Yangzhou University, Yangzhou, China; ^3^Biotechnology Division, CSIR-Institute of Himalayan Bioresource Technology, Palampur, India; ^4^BGI-Shenzhen, Shenzhen, China; ^5^Joint International Research Laboratory of Agriculture and Agri-Product Safety, The Ministry of Education of China, Yangzhou University, Yangzhou, China

**Keywords:** circRNAs, tomato, cold stress, miRNA sponge, post-transcriptional regulation

## Abstract

Abiotic stress adversely inhibits the growth and development of plants, by changing the expression of multiple genes. Circular RNAs (circRNAs), as a class of non-coding RNAs, function in transcriptional and posttranscriptional regulation. Yet, the involvement of circRNAs in abiotic stress response is rarely reported. In this study, the participation and function of circRNAs in low-temperature (LT)-induced stress response were investigated in tomato leaves. We generated genome-wide profiles of circRNAs and mRNAs in tomato leaves grown at 25°C room temperature (RT) and 12°C LT. Our results show that 1,830 circRNAs were identified in tomato leaves in both RT and LT treatments, among which 1,759 were differentially induced by the LT treatment. We find that the identified circRNAs are mainly located at exons of genes, but less distributed at introns of genes or intergenic regions. Our results suggest that there are 383 differentially expressed circRNAs predicted to function as putative sponges of 266 miRNAs to target 4,476 mRNAs in total. Moreover, Gene Ontology (GO) and Kyoto Encyclopedia of Genes and Genomes (KEGG) analysis assays indicate that multiple pathways were enriched in both differentially expressed genes induced by LT and parental genes of differentially expressed circRNAs induced by LT, revealing the key functions of circRNAs and the corresponding targeted genes in response to LT stress. Our results suggest that circRNAs may be involved in regulating metabolism (i.e., carbohydrate, amino acid, lipid, and energy), signal transduction, and environmental adaptation-related pathways and that these circRNAs were predicted to regulate the expression of transcription factors, genes in signal transduction pathways, and genes related to the Ca^2+^ channel through targeting the corresponding proteins, such as WRKY, NAC, cytochrome P450, and calmodulin binding protein. Taken together, our study uncovers that multiple circRNAs are isolated and differently regulated in response to LT stress and provides the resource and potential networks of circRNA–miRNA–mRNA under LT stress for further investigations in tomato leaves.

## Introduction

Most of the genomes in eukaryotes generate RNA transcripts that do not code for proteins. Short non-coding RNAs (smaller than 200 nt) including small interfering RNAs (siRNAs), microRNAs (miRNAs), and PIWI-interacting RNAs (piRNAs) are well studied in the regulation of gene expression (Zhang et al., 2018). Long non-coding RNAs (lncRNAs) regulate multiple gene expressions that are enriched in various biological processes in eukaryotes (Zaynab et al., 2018). The lncRNAs are the key regulators of chromatin remodeling and genome pattern, RNA stabilization, and transcription regulation. The lncRNAs are well known to function as important regulators in response to abiotic stress response in plants. Circular RNAs (circRNAs) are recently discovered endogenous non-coding RNAs (Salzman et al., 2012; Memczak et al., 2013; Wang et al., 2014). The circRNAs are derived from linear precursor mRNAs, which can be generated from circularization of lariat-driven and intron-pairing-driven, alternative splicing, and protein factors associated circulation (Zhang et al., 2014; Starke et al., 2015; Chen, 2016; Liu J. et al., 2017). According to the original positions in pre-mRNAs, the circRNAs are classified into three groups: exonic, intronic, and intergenic circRNAs (Zhang et al., 2013; Jeck and Sharpless, 2014). Although circRNAs were firstly discovered in humans by electron microscopy nearly three decades ago, circRNAs had been previously considered as a rare existence in nature and as transcriptional noise or artifacts for a long time (Salzman, 2016). The circRNAs are generated in the nucleus and then transferred to the cytoplasm (Hansen et al., 2013; Memczak et al., 2013; Jeck and Sharpless, 2014). CircRNAs are highly abundant and stable, compared to their linear counterparts (Hansen et al., 2011; Salzman et al., 2012, 2013; Jeck et al., 2013; Memczak et al., 2013), suggesting their potential biological significance. Recent studies reveal that circRNAs are conserved among species and are mainly expressed in a cell-, tissue-, and developmental stage-dependent manner (Jeck et al., 2013; Memczak et al., 2013; Salzman et al., 2013; Szabo et al., 2015; Venø et al., 2015).

With the development of high-throughput sequencing methods, circRNAs have been isolated in bacteria (Danan et al., 2012), fungi (Wang et al., 2014), animals (Zhang et al., 2013; Ashwal-Fluss et al., 2014), and humans (Memczak et al., 2013). In plants, circRNAs were firstly discovered in *Arabidopsis thaliana* (Wang et al., 2014; Ye et al., 2015; Conn et al., 2017). After that, circRNAs have been gradually identified in rice (Lu et al., 2015; Ye et al., 2015), barley (Darbani et al., 2016), kiwi (Wang Z.P. et al., 2017), and wheat (Wang et al., 2017b), indicating that circRNAs commonly exist in monocots and dicots. Compared to the circRNAs identified in animals, plant circRNAs likely are shorter in the sequences of the flanking introns (Ye et al., 2015). Moreover, plant circRNAs display the feature of regulating multiple biological pathways. The fact that circRNAs and their linear forms might negatively regulate the posttranscriptional level of their parental gene was further confirmed by overexpression of Os08circ16564 in rice (Lu et al., 2015). Six differentially expressed circRNAs were identified in response to dehydration stress in wheat (Wang et al., 2017b). Recent studies suggest that circRNAs could function as miRNA sponges and thereby impair miRNA-directed gene silencing (Hansen et al., 2013; Memczak et al., 2013). These studies hint that circRNAs plausibly respond to abiotic stress through regulating the expression of stress-related genes.

Cold stress is a major environmental challenge that largely influences the growth and development of plants (Agarwal et al., 2006; Chinnusamy et al., 2006; Jiang et al., 2013). Cold stress is classed into chilling stress (0–20°C) and freezing stress (<0°C; Chinnusamy et al., 2010); low temperature (LT) greatly influences crop productivity, especially in chilling-sensitive crops (Zhou et al., 2011). Cold stress tolerance increases in plants after previous exposure to LT. This mechanism of adaptation is usually called cold acclimation, which results in numerous changes in gene expression (Steponkus, 1984; Guy, 1990; Thomashow, 1999). It has been shown that two calcium/calmodulin-regulated receptor-like cytoplasmic kinases (RLCKs), CRLK1, and CRLK2, positively regulate freezing tolerance (Yang et al., 2010a, b; Zhao C. et al., 2017). Recently, a plasma membrane-localized RLCK, cold-responsive protein kinase 1 (CRPK1), was reported to negatively regulate freezing tolerance (Liu Z. et al., 2017). Recent studies show that the two non-membrane proteins located in the nucleus and cytosol, including the histone variant H2A.Z and the photoreceptor phytochrome B (phyB), probably function as temperature sensing (Kumar and Wigge, 2010; Jung et al., 2016; Legris et al., 2016). The reactive oxygen species (ROS) induced by the cold may activate a mitogen-activated protein kinase cascade (AtMEKK1-AtMKK2-AtMPK4/6) that enhances the tolerance to freezing and other abiotic stresses (Kovtun et al., 2000; Teige et al., 2004). Although circRNAs have been identified and are reported to be involved in tomato fruit ripening, coloration, pigment accumulation, and ethylene pathway; chilling injury on fruits; and *Phytophthora infestans* infection in tomato (Zuo et al., 2016; Tan et al., 2017; Wang et al., 2017a; Yin et al., 2018; Zhou et al., 2018b; Hong et al., 2020), the roles of circRNAs in response to abiotic stresses (i.e., LT) in tomato plants are still unknown.

In this study, we used high-throughput sequencing to identify genome-wide mRNAs and circRNAs in response to LT stress in tomato leaves. We identified a total of 1,830 circRNAs, among which 1,759 were differentially regulated under LT stress. Of these circRNAs, 383 differentially expressed circRNAs were identified to function as putative sponges of 266 miRNAs to target 4,476 mRNAs. Gene Ontology (GO) and Kyoto Encyclopedia of Genes and Genomes (KEGG) annotations for parental genes of circRNAs and mRNAs revealed that circRNAs and mRNAs were involved in regulating metabolism (amino acid, carbohydrate, lipid, and energy), signal transduction, and environmental adaptation. Furthermore, these circRNAs were predicted to regulate the genes related to transcription and the Ca^2+^ channel by targeting the associated proteins such as NAC, MYB, WRKY, and calmodulin-binding protein. Hence, our study improves the understanding on the function of circRNAs in cold response in tomato plants.

## Materials and Methods

### Plant Materials and Cold Stress Treatment

The wild-type tomato (*Solanum lycopersicum*) “cv 1479” were used to perform LT stress assays. The seeds of tomato were germinated at trays with vermiculite substrate, and then the seedlings were transferred to pots. The well-grown and 2-week-old seedlings were divided into two groups (*n* = 12). The two groups were treated with room temperature (25°C/22°C, day/night, 14-h photoperiod, referred to as RT) and low temperature (15°C/12°C, day/night, 14-h photoperiod, referred to as LT), with 60–70% relative humidity for 3 weeks, respectively. After the treatments, the fifth leaf of each plant was harvested and frozen immediately in liquid nitrogen for further analysis. Three biological replicates were performed, and the fifth leaves from four individual plants were mixed and referred to as one biological replicate.

### Library Preparation for circRNA-seq and mRNA-seq

All the sequencing was performed at BGI, China. The total RNAs were isolated from the leaves of tomato plants using the TRIzol reagent (Invitrogen, United States) according to the manufacturer’s instruction. The concentration and purity of total RNAs were checked by the NanoDrop ND-1000 spectrophotometer (NanoDrop Technologies, United States). Ribosomal RNA (rRNA) was removed by Ribo-Zero rRNA Removal Kits (Illumina, United States). The RNA integrity was checked by the Agilent 2100 Bioanalyzer Lab-on-Chip system (Agilent Technologies, United States). The RNAs were further incubated at 37°C for 1 h for the digestion with RNase R (Epicentre, WI, United States). The purified RNAs were used as templates for the construction of cDNA libraries according to the protocol for the mRNA-seq sample preparation kit (Illumina, United States). The clustering of samples was performed on a cBot Cluster Generation System using TruSeq PE Cluster Kit v3-cBot-HS (Illumina, United States) following the manufacturer’s procedure. The paired-end sequencing was detected with the Illumina HiSeq X Ten platform (BGI, Wuhan, China).

### Read Alignment and Identification of circRNA

The clean reads of three biological replicates in RT and LT samples were assembled into two data libraries. The low-quality reads, including unknown bases greater than 5%, those containing more than 50% bases with *Q* ≤ 20 ploy-N, and adaptor sequences, were removed. Q20, Q30, and GC contents were calculated. Reads from every sample were mapped to the *S. lycopersicum* reference genome^1^ using TopHat version 2.1.1 (Kim et al., 2013). The SAM files were then treated by CIRI (v2.05; Gao et al., 2015) and find_circ (v1.2) with genomic annotations from International Tomato Annotation Group 2.4 (ITAG2.4) to identify circRNAs. The CIRI software scans SAM files twice and then collects the related information to isolate the circRNAs. CIRI exactly detects the junction reads with PCC signals that reflect the candidate of a circRNA during the first scanning of SAM alignment. Preliminary filtering is performed by the paired-end mapping (PEM) and GT-AG splicing signals for the junctions. When the clustering of junction reads and recording of each circRNA candidate are finished, CIRI scans the SAM alignment one more time to detect additional junction reads and simultaneously performs further filtering to remove the false-positive candidates generated from the reads incorrectly mapped to the homologous genes or repetitive sequences. The circRNA calling was done by using find-circ software, meeting the following conditions: GU/AG, on the sides of splice site; clear breakpoint; two mismatches; appearance of breakpoint in the position within 2 nucleotides (nt); more than two reads supporting the junction; a score of blasting to the right position of short sequence that is at least 35 higher than blasting to the other positions. Finally, identified circRNAs are outputted with annotation information.

### Expression Level Analysis of circRNAs

The expression levels of circRNAs were calculated by the number of junction reads at both ends of the circRNAs identified by CIRI (v2.0.5) and find_circ (v1.2) tools. Since two software, CIRI and find_circ, were used for the prediction, the final junction reads were averaged. We used the DESeq2 algorithm to detect differentially expressed circRNAs. Genes with an absolute value of log2 (fold change) ≥ 1 and *P* value < 0.05 found by DESeq2 were defined as differentially expressed genes. The expression levels of each circRNA identified in three biological replicates was averaged to get the final expression value of the corresponding circRNA.

### circRNA Annotation and GO Enrichment

The function of genes was annotated according to the following databases: Swiss-Prot (a manually annotated and reviewed protein sequence database), NCBI non-redundant protein sequences, KOG/COG (Clusters of Orthologous Groups of proteins), Pfam (protein family), KEGG, and GO. The corresponding parental genes of differentially expressed circRNAs were collected. The GO enrichment assays were performed using topGO R packages.

### Prediction for circRNA–miRNA–mRNA Relationships

The miRNA binding sites of tomato circRNAs upon the alignment against miRBase21.0^2^ were predicted by miRanda (3.3a) and TargetScan (V7.0; Kozomara and Griffiths-Jones, 2014). The FASTA-formatted sequences of predicted target miRNAs were detected by a website^3^ to find the corresponding target coding genes (*S. lycopersicum*, transcript, cDNA library, version 2.4). All the results were visualized by Cytoscape (v3.5.0) to clearly display the relationships among the circRNA–miRNA–mRNA (Shannon et al., 2003).

## Results

### Identification of circRNAs in Tomato Leaves

In order to isolate potential circRNAs in tomato leaves, polyA-depleted RNA libraries from 5-week-old tomato leaves grown at RT and LT were constructed and sequenced by the Illumina HiSeq X Ten platform. In total, about 200 million paired-end raw reads were generated from samples in RT and LT (Supplementary Table S1). Low-quality reads were firstly filtered, and clean reads were then mapped to reference genome *S. lycopersicum* (SL3.0), resulting in about 160 million unique mapped reads in RT and LT samples used for further analysis (Supplementary Table S1).

**FIGURE 1:**
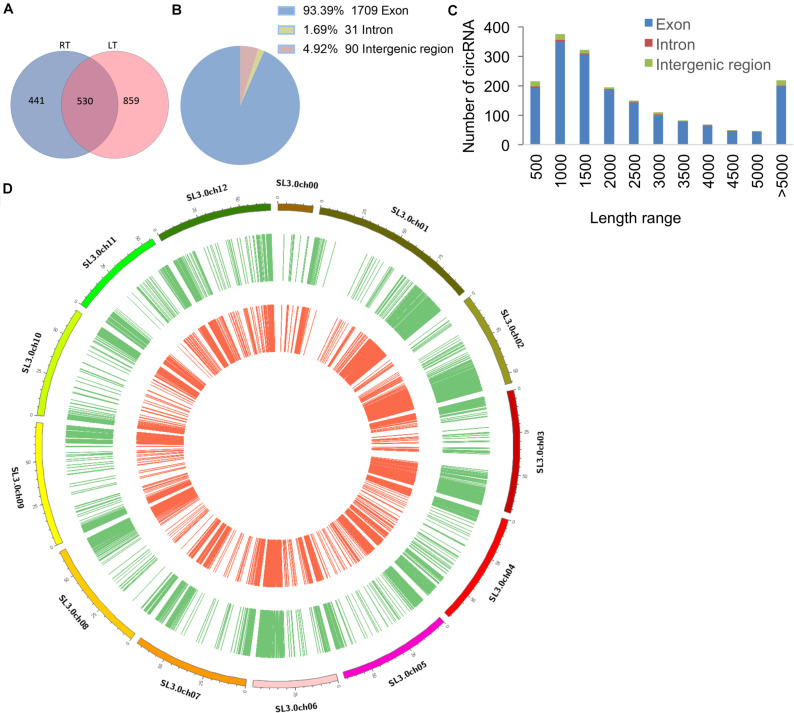
Characterization of tomato circRNAs in different temperature treatments. **(A)** Venn diagram shows the number and distribution of detected circRNAs in tomato samples at room temperature (RT) and low temperature (LT). The chromosomes of *S. lycopersicum* (version SL3.0) are displayed. **(B)** The pie chart represents the classification of the circRNAs according to the location of the genomic region. **(C)** The histogram shows the number of circRNAs in different ranges of length. **(D)** The distribution of all the identified circRNAs in samples of RT and LT (*n* = 1,830). circRNAs identified in tomato leaves at LT treatment are displayed in green, and circRNAs identified at RT treatment are displayed in red.

There were 1,830 circRNAs identified, 530 of which existed in both RT and LT samples (Figure 1A and Supplementary Table S2). Among the identified circRNAs, 1,709 (93.39%) were exonic circRNAs, 90 (4.92%) were intergenic regions (intergenic circRNAs), and 31 (1.69%) were intronic circRNAs (Figure 1B). The length-dependent count analysis showed that more than half of the circRNAs are shorter than 2 kb (Figure 1C). The circRNAs identified in RT treatment and LT treatment displayed similar expression patterns on tomato chromosomes (Figure 1D). These results indicated that circRNAs in tomato leaves were generated from different genomic regions and chromosomes, showing the characterization of various lengths.

### LT Treatment Induces the Expression of circRNAs in Tomato

**FIGURE 2:**
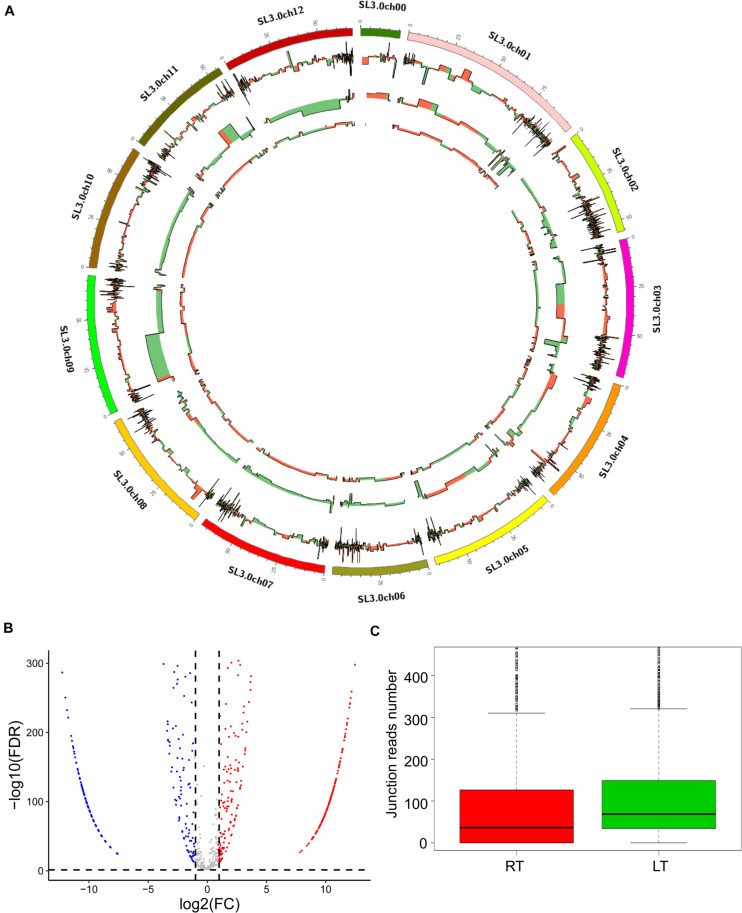
LT treatment induces the expression of various circRNAs in tomato leaves. **(A)** Distribution of differentially expressed mRNA, miRNA, and circRNA on the tomato chromosomes. The first circle (from outside to inside) represents chromosomes of *S. lycopersicum* (version SL3.0), the second circle represents mRNA expression, the third circle represents miRNA expression, and the fourth circle represents circRNA expression. Red indicates upregulation, and green indicates downregulation. **(B)** The expression profile of the circRNAs in the leaves grown at RT and LT. Red dots represent the upregulated circRNAs, blue dots represent the downregulated circRNAs, and gray dots represent the unchanged circRNAs. **(C)** The box plot shows back-splice junction reads at RT and LT treatments.

To test the induction of LT on the expression of circRNAs in tomato leaves, we compared the expression of circRNAs between the treatments of RT and LT. We found that 1,759 circRNAs were significantly expressed in the LT treatment, compared to RT, and that 1,115 and 644 circRNAs were upregulated and downregulated, respectively, (Figures 2A,B and Supplementary Table S2). The distribution of circRNAs, mRNA, and miRNA on chromosomes was displayed (Figure 2A), indicating a high association among the circRNAs, miRNA, and the targeted mRNA. In terms of the junction read number, the expression of circRNAs in samples at LT were higher than that at RT by boxplot representation (Figure 2C). These results suggest that LT treatment greatly induced the expression of circRNAs in tomato leaves.

### Biological Function Analysis of circRNAs Induced by LT

**FIGURE 3:**
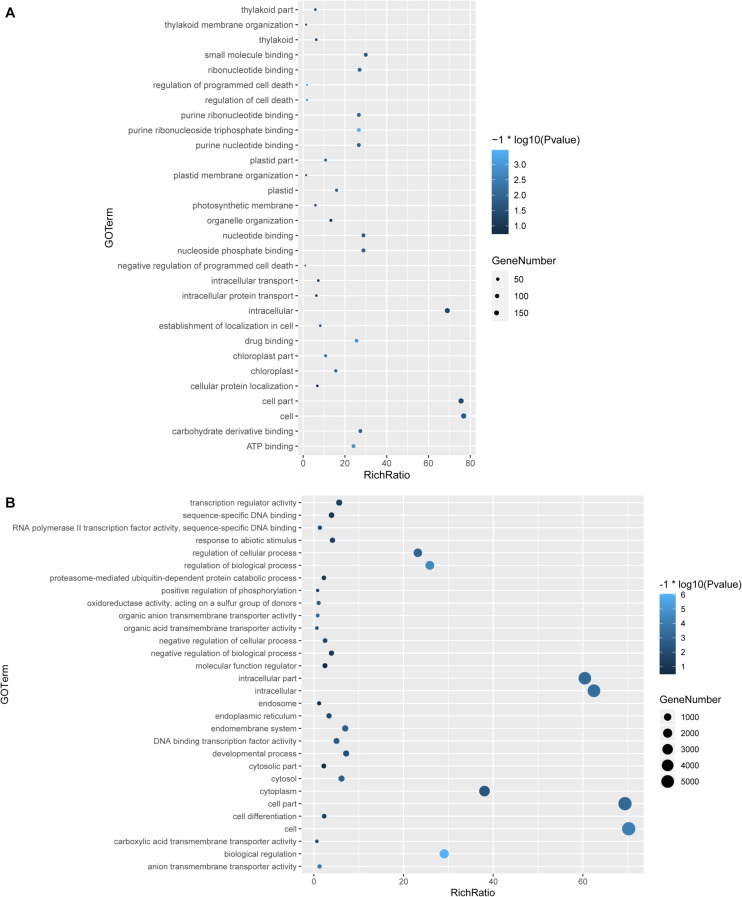
GO analysis of genes and circRNAs induced by LT treatment. **(A)** GO analysis of parental genes of differentially expressed circRNAs under LT treatment. **(B)** GO analysis of differentially expressed genes under LT treatment.

To understand the function of the circRNAs, GO and KEGG analyses were performed in the parental genes of the circRNAs and in the differentially expressed genes induced by LT. By GO analysis, we found that multiple pathways were enriched in the parental genes of the circRNAs induced by LT treatment; among these pathways, nucleotide binding and purine binding were significantly enriched (Figure 3A). Pathways related to abiotic stress, DNA binding, regulation of biological process, and transcription regulator activity were enriched in the differentially expressed genes induced by LT treatment (Figure 3B).

**FIGURE 4:**
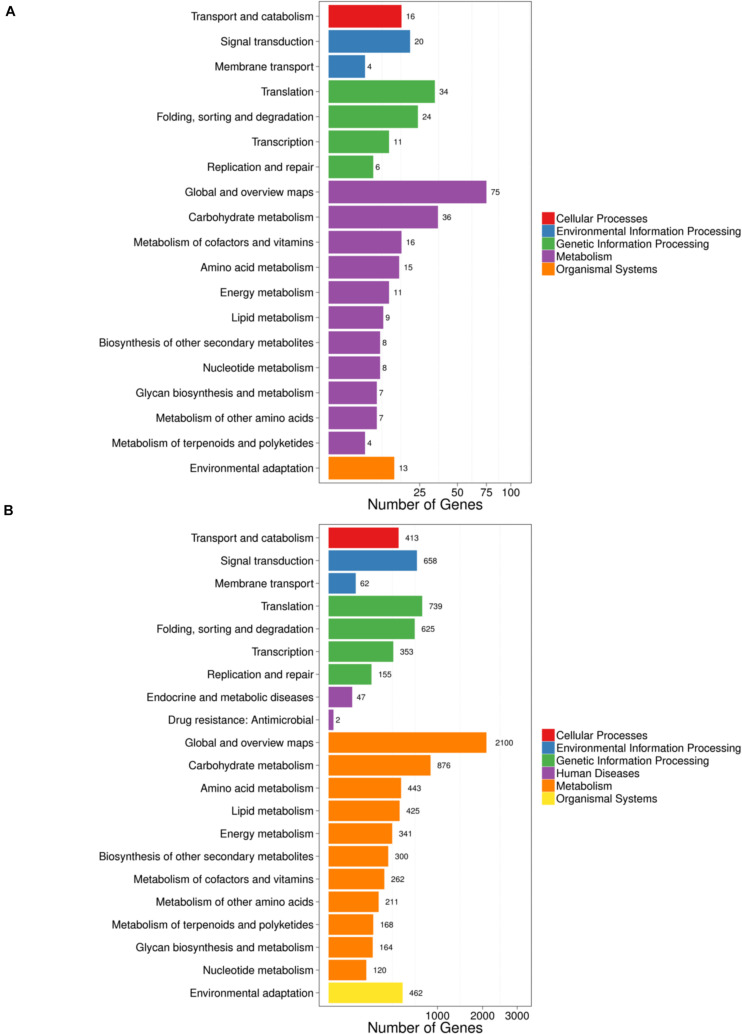
KEGG analysis of genes and circRNAs induced by LT treatment. **(A)** KEGG analysis of parental genes of circRNAs differentially expressed under LT treatment. **(B)** KEGG analysis of differentially expressed genes under LT treatment. The rectangles indicate the mRNAs. The ellipses indicate the circRNAs. Red, upregulated; green, downregulated.

Kyoto Encyclopedia of Genes and Genomes pathway analysis showed that the parental genes of circRNAs (Figure 4A) and LT-induced genes (Figure 4B) were mostly enriched in transport and catabolism, signal transduction, metabolism (carbohydrate, amino acid, lipid, energy, nucleotide, terpenoids, and polyketides), and environmental adaptation. The results suggested that circRNAs likely participated in cellular processes and metabolic processes. Notably, the specific and detailed function of circRNAs needs to be investigated in further studies.

### circRNAs Function As Putative miRNA Sponges to Regulate Transcription of Genes

**FIGURE 5:**
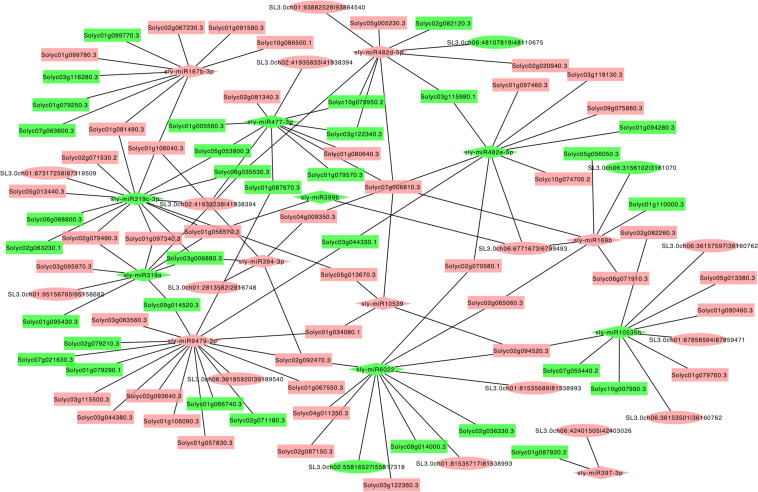
The predicted regulatory networks regulated by circRNAs and the corresponding targeted genes during LT stress response in tomato leaves. The diamonds indicate miRNAs; the ellipses indicate circRNAs; the rectangles indicate targeted genes. Red indicates upregulation, and blue indicates downregulation.

The circRNAs function to target miRNAs and inhibit the activity of the miRNAs. The miRNAs have been shown to take part in several physiological processes by controlling the expression of genes in plants. To reveal the role of circRNAs in response to LT in tomato plants, the miRNA targets of circRNAs were predicted, and the circRNA–miRNA–mRNA networks were generated. Among these identified circRNAs in RT and LT samples, 383 differentially expressed circRNAs were predicted to function as the putative sponges of 266 miRNA to further regulate 4,476 targeted mRNAs (Figure 5 and Supplementary Table S3). We found that targeted mRNAs included various genes that were presented in transcription and signaling transduction pathways, such as several transcription factor families and proteins including calcium (Ca^2+^) channels and receptor-like kinases (RLKs), cytochrome P450, and E3 ubiquitin-protein ligase (Supplementary Table S4). Many miRNAs targeted transcription factors, including NAC, MYB, WRKY, WD40, zinc finger transcription factor, bHLH, and Hsf (Supplementary Table S4). For example, miR-9479-3p targeted cytochrome P450, MYB, and bHLH; miR-477-3p targeted bHLH, calmodulin, WRKY, and MYB (Supplementary Table S4). These results further confirm that circRNAs are critical in regulating transcription by playing as miRNA sponges in response to LT treatment in tomato leaves.

## Discussion

The circRNAs are a novel class of non-coding RNAs that are commonly explored. Various circRNAs are highly expressed and evolutionarily conserved (Salzman, 2016). Recently, plant circRNAs have been identified and reported in *Arabidopsis* (Ye et al., 2015), barley (Darbani et al., 2016), rice (Ye et al., 2015), tomato (Zuo et al., 2016), soybean (Zhao W. et al., 2017), wheat (Wang et al., 2017b), cotton (Zhao T. et al., 2017), kiwi (Wang Z.P. et al., 2017), potato (Zhou et al., 2018b), maize (Chen et al., 2018), and orange (Zeng et al., 2018). The identification of circRNAs accelerates the understanding of the functions of circRNAs in different biological processes in plants. Cold stress is a great challenge to plants, inducing loss of yield and quality damage to crops. Moreover, cold response in plants is a complex process, including the processes of inducing expression changes of the calcium channel protein, metabolism-related proteins, and multiple transcription factors (An et al., 2018; Sun et al., 2018; Xie et al., 2018; Fürtauer et al., 2019; Liu et al., 2019; Gong et al., 2020). Epigenetic regulations, including DNA methylation, histone modifications (variants), and non-coding RNAs are essential to gene expression (Zhang et al., 2018; Nie et al., 2019). Hence, understanding the epigenetic regulation in response to cold stress in plants is of interest. So far, the roles of circRNAs in the processes of cold tolerance are not well known, especially in thermophilic vegetable crops, such as in tomato plants (Camejo et al., 2005). In this study, we report the isolation of 1,830 circRNAs in the leaves of 5-week-old tomato seedlings and find 859 circRNAs specifically expressed in response to LT treatment (Figure 1A). We characterize the whole-genome-wide pattern and the distribution on chromosomes of tomato circRNAs (Figures 1D, 2A). In addition, we clearly map the relationship among differentially expressed mRNAs, miRNAs, and circRNAs under LT treatment (Figure 2A). These sequencing data provide much information for further studies on the function of circRNAs in tomato plants, especially on the investigations of tomato plants responding to abiotic stress.

Circular RNAs are classified into three groups: exonic circRNAs, intronic circRNAs, and intergenic circRNAs (Jeck and Sharpless, 2014). In our study, we find that 1,709, 90, and 31 circRNAs were exonic, intergenic, and intronic circRNAs, respectively, (Figure 1B). These results are consistent with the previous reports in *Arabidopsis*, rice, and tomato (Lu et al., 2015; Ye et al., 2015; Zuo et al., 2016; Wang et al., 2017a; Pan et al., 2018; Zhou et al., 2018b), suggesting that the circRNA-calling method in our study is reliable. Meanwhile, circRNAs were largely distributed at chromosome 1, consistent with results of previous studies in tomato (Zuo et al., 2016; Wang et al., 2017a; Yin et al., 2018; Zhou et al., 2018a). These results, as well as our methods in identification of circRNAs, contribute to the progress of the development of the field of plant circRNAs. In tomato, most studies on circRNAs are mainly performed in fruit (Zuo et al., 2016; Wang et al., 2017a; Yin et al., 2018; Zhou et al., 2018a). A previous study reported that circRNAs show ripening-dependent characterizations and that the parental genes of the circRNAs with significantly different expression levels were mainly involved in metabolic, cellular, and single-organism processes and catalytic and binding activities (Zhou et al., 2018a). It has been shown that several circRNAs were specific in tomato fruits at different developmental stages, which enriches the number of plant circRNAs involved in fruit coloration and ripening (Zhou et al., 2018a). Besides, circRNAs were predicted to be involved in transcription regulation through targeting transcription factors such as ethylene-responsive transcription factor (ERF), squamosa promoter binding-like protein (SBP), and myeloblastosis (MYB) proto-oncogene protein (Yin et al., 2018). These results uncover the role of circRNAs in fruit ripening and isolate the plausible circRNAs in tomato fruits. The circRNAs are likely to regulate fruit ripening by controlling metabolism adaptation, hormone content, and photosynthesis related pathways (Yin et al., 2018). Unlike the investigation of circRNAs in tomato fruits, here, we report the role of circRNAs in LT stress response in tomato leaves, generating genome-wide profiles of circRNAs and mRNAs in tomato leaves with LT treatment. Our results show that 1,759 circRNAs were LT dependent in tomato leaves. The numbers of circRNAs identified in our study is different to that identified in the fruits (Yin et al., 2018; Zhou et al., 2018a), indicating that the abundance and the kind of circRNAs in different tissues are various. Under LT stress treatment, the parental genes of the differentially expressed circRNAs in tomato leaves are enriched in the pathways of regulating metabolism (i.e., carbohydrate, amino acid, lipid, and energy), signal transduction, and environmental adaptation, and these circRNAs were predicted to regulate the expression of genes including transcription factors, signal transduction pathways, and Ca^2+^ channel through targeting the corresponding proteins, such as WRKY, NAC, cytochrome P450, and calmodulin-binding protein. Thus, our results connect the relationship between circRNAs and abiotic stress response in tomato leaves, providing a useful perspective for further understanding the mechanisms of abiotic stress response (i.e., LT) in tomato plants.

Studies have shown that photosynthetic light reactions and the central carbohydrate metabolism are immediately reprogrammed to prevent any imbalances that would cause the production of ROS, cell damage, and cell death in response to changes of temperature (Huner et al., 1998; Hu et al., 2008). Changing temperature affects photosynthesis immediately (Hu et al., 2006; Nägele et al., 2011; Nie et al., 2013). In this study, GO enrichment analysis of parental genes of LT-induced circRNAs indicates that various photosynthesis-related pathways were enriched in LT treatment, such as the thylakoid part, thylakoid membrane organization, regulation of programmed cell death, photosynthetic membrane, and chloroplast (Figure 3A). These results suggest that LT-induced circRNAs likely function on regulating photosynthesis progress in response to LT, consistent with the importance of photosynthesis in response to abiotic stress (Nie et al., 2013; Gururani et al., 2015). It is meaningful to test whether circRNAs could regulate the photosynthesis rate and thus further regulate the biomass of tomato plants in the future, which is conveniently confirmed by the genetic mutants by CRISPR. Unlike the GOs enriched in LT-treated tomato leaves, chilling injury treatment on tomato fruits could induce some circRNAs that were predicted to regulate chilling-responsive processes, such as redox reaction, arginine and polyamine metabolism, cell wall degradation, heat and cold shock protein, energy metabolism, and metabolism of jasmonic acid and abscisic acid (Zhou et al., 2018a). Hence, LT-induced circRNAs display tissue-dependent properties and functions.

In rice and *Arabidopsis*, the expression levels of most circRNAs and their corresponding parental genes were positively correlated (Ye et al., 2015). It has been shown that carbohydrates are essential for the metabolic reprogramming during cold acclimation (Fürtauer et al., 2019); freezing tolerance in plants is dependent on lipid remodeling at the outer chloroplast membrane (Moellering et al., 2010). In this study, by KEGG analysis, we found that the parental genes of circRNAs (Figure 4A) and differentially expressed genes (Figure 4B) share lots of pathways, such as transport and catabolism, signal transduction, metabolism (carbohydrate, amino acid, lipid, energy, nucleotide, terpenoids, and polyketides), and environmental adaptation, suggesting circRNAs and mRNA are coordinately regulated in plants when responding to cold stress. Given the high correlation between circRNAs and the corresponding parental genes, it is possible to investigate the detailed genetic function of these parental genes in the signaling pathway in response to LT stress in further studies.

Several studies reported that circRNAs play important roles in miRNA function and transcriptional control by competing with endogenous RNAs or positive regulators on their parental genes (Hansen et al., 2013; Memczak et al., 2013; Ye et al., 2015). Following the published methods of bioinformatics analysis (Wu et al., 2013; Ye et al., 2014), we found that 383 circRNAs were potential sponges of 266 miRNAs, which target 4,476 mRNAs (Supplementary Table S3). These targeted genes of miRNAs include genes participating in signaling transduction pathways, such as calcium (Ca^2+^) channels and RLKs, cytochrome P450, and E3 ubiquitin-protein ligase (Supplementary Table S4). Moreover, multiple miRNAs target transcription factors such as NAC, MYB, WRKY, WD40, zinc finger transcription factor, bHLH, and HSF (Supplementary Table S4). For example, we find that miR-9479-3p is predicted to target cytochrome P450, MYB, and bHLH; miR-477-3p is predicted to target bHLH, calmodulin, WRKY, and MYB (Supplementary Table S4), indicating that circRNAs likely regulate the expression of transcriptional factors through targeting miRNAs. Although the putative miRNA-binding sites of circRNAs have been identified, genetic evidence for their biological function in tomato leaves needs further investigations. Our results provide a resource to uncover the functions of circRNAs in response to abiotic stress in tomato leaves. It should be noted that further studies on the networks of circRNA–miRNA–mRNA are essential to fully understand the mechanisms of cold stress tolerance mediated by circRNAs.

## Data Availability Statement

The sequencing data was uploaded to NCBI and the accession Nos. are SRR12489163–SRR12489168.

## Author Contributions

XY, WZ, and W-FN designed the research. XY, YL, HZ, and JW performed the research. XY, YL, SX, and W-FN analyzed and interpreted data. XY, YL, and W-FN wrote the manuscript. XY, W-FN, and GZ contributed to the manuscript editing. All authors contributed to the article and approved the submitted version.

## Conflict of Interest

SX was employed by company BGI-Shenzhen. The remaining authors declare that the research was conducted in the absence of any commercial or financial relationships that could be construed as a potential conflict of interest.

## Footnotes

1 ftp://ftp.solgenomics.net/tomato_genome/assembly/build_3.00/, version SL3.0.

2 http://www.mirbase.org/

3 http://plantgrn.noble.org/psRNATarget/

## Supplementary Material

Supplementary Table 1 The summary of sequencing quality.

Supplementary Table 2 The circRNAs identified in tomato plants.

Supplementary Table 3 The prediction of miRNA-circRNA-mRNA networks.

Supplementary Table 4 The predicted targets of miRNA and the annotation of the corresponding genes.
